# Wallerian degeneration: the innate-immune response to traumatic nerve injury

**DOI:** 10.1186/1742-2094-8-109

**Published:** 2011-08-30

**Authors:** Shlomo Rotshenker

**Affiliations:** 1 Dept. of Medical Neurobiology, IMRIC, Hebrew University, Faculty of Medicine, Jerusalem, Israel

**Keywords:** Wallerian degeneration, macrophage, phagocytosis, cytokine, myelin

## Abstract

Traumatic injury to peripheral nerves results in the loss of neural functions. Recovery by regeneration depends on the cellular and molecular events of Wallerian degeneration that injury induces distal to the lesion site, the domain through which severed axons regenerate back to their target tissues. Innate-immunity is central to Wallerian degeneration since innate-immune cells, functions and molecules that are produced by immune and non-immune cells are involved. The innate-immune response helps to turn the peripheral nerve tissue into an environment that supports regeneration by removing inhibitory myelin and by upregulating neurotrophic properties. The characteristics of an efficient innate-immune response are rapid onset and conclusion, and the orchestrated interplay between Schwann cells, fibroblasts, macrophages, endothelial cells, and molecules they produce. Wallerian degeneration serves as a prelude for successful repair when these requirements are met. In contrast, functional recovery is poor when injury fails to produce the efficient innate-immune response of Wallerian degeneration.

## Introduction

Traumatic injury to nerves in the PNS (peripheral nervous system) results in the loss of neural functions. Repair is achieved through regeneration of severed axons and reinnervation of target tissues. Successful functional recovery depends on the ensemble of cellular and molecular events that develop distal to lesion sites all the way towards denervated target tissues. Those represent the PNS response to traumatic nerve injury and are termed collectively Wallerian degeneration after Waller [1].

Numerous studies have been carried out since Waller first documented his findings. They provide essential, yet incomplete understanding of the mechanisms that control Wallerian degeneration and how those may be influenced to provide grounds for best functional recovery. Wallerian degeneration has been reviewed in recent years; e.g. [2-6] and additional publications that are cited throughout the text.

This review focuses on the cellular and molecular events that highlight Wallerian degeneration as the innate-immune response of the PNS to traumatic nerve injury (e.g. recruitment of macrophages, phagocytosis of degenerated myelin, and production of cytokines and chemokines). Special attention is given to the orchestration of these events with respect to their timing and magnitude, and to the identity of the cells that produce them. Timing differs between species (see below). Therefore, it is important to consider which animal model was used when analyzing and integrating data. Those that will be most discussed here are wild-type and mutant Wld^s ^mice, which respectively display "normal Wallerian degeneration" and delayed "slow Wallerian degeneration". Further, the coordination between cellular and molecular events of Wallerian degeneration that follow crush injuries may differ from those that follow cut injuries. The connective tissue sheath of peripheral nerves does not tear apart after crush but does so after complete transection. Therefore, it is difficult to ascertain that all axons are severed by crushing. Additionally, severed axons regenerate readily after crush but not after transection. Consequently, the cellular and molecular events of Wallerian degeneration may be altered by the regenerating axons (see below). Therefore, the nature of the injury must also be considered.

The term Wallerian degeneration has been adopted to describe events that follow traumatic injury to CNS (central nervous system) axons (e.g. spinal cord injury). However, Wallerian degeneration in PNS and CNS differ with respect to the types of cells involved (e.g. Schwann cells and macrophages in PNS versus oligodendrocytes and microglia in CNS) and outcome (e.g. removal of degenerated myelin during PNS Wallerian degeneration but not during CNS Wallerian degeneration). Therefore, it may be useful to use the terms PNS Wallerian degeneration and CNS Wallerian degeneration to avoid confusion when both are discussed. Further, the term Wallerian degeneration is sometimes used to define events that develop during PNS neuropathies without trauma (e.g. inherited demyelinating diseases). However, those differ from injury-induced Wallerian degeneration, which may lead to confusion. The term Wallerian degeneration that is used in this review refers to injury-induced PNS Wallerian degeneration unless otherwise specified.

### Traumatic injury to peripheral nerves, Wallerian degeneration and functional recovery

**Figure 1:**
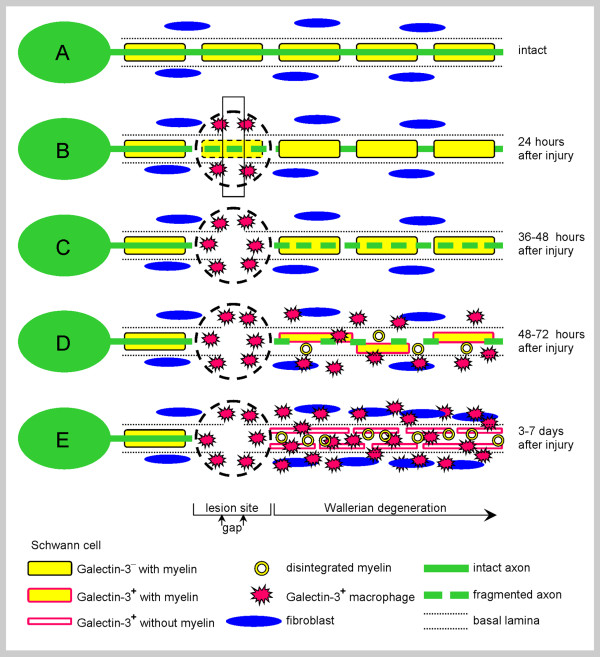
**Intact and injured PNS nerves**. A schematic representation of some of the cellular characteristics of (A) intact and (B through E) injured PNS nerves that undergo normal Wallerian degeneration. (A) Intact myelinating Schwann cells enwrap an intact axon and fibroblasts are scattered between nerve fibers. (B) Traumatic injury produces immediate tissue damage at the lesion site (marked by a circle), a gap (rectangle) may be formed between the proximal and distal nerve stumps, and Galectin-3/MAC-2^+ ^macrophages accumulate at the lesion site within 24 hours after the injury. (C) Destruction of axons is detected during normal Wallerian degeneration 36 hours after the injury. (D) Recruitment of Galectin-3/MAC-2^+ ^macrophages, myelin disintegration, and Galectin-3/MAC-2 expression by Schwann cells begin 48 to 72 hours after injury during normal Wallerian degeneration. (E) Galectin-3/MAC-2^+ ^macrophages and Schwann cells scavenge degenerated myelin during normal Wallerian degeneration, and Schwann cells further proliferate and form Bünger bands.

**Figure 2:**
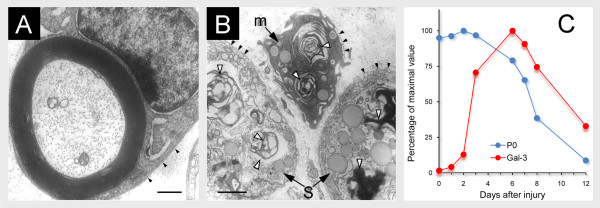
**Intact axon, normal Wallerian degeneration, and kinetics of myelin clearance and Galectin-3/MAC-2 expression during normal Wallerian degeneration**. (A) A Schwann cell that is surrounded by basal lamina (arrow heads) forms a myelin sheath around an intact axon; Bar 1 μm. (B) Axons are not detected 7 days after the injury, and Schwann cells (S) and a macrophage (m), which are situated within basal lamina sheaths (dark arrow heads), contain myelin fragments and lipid droplets in their cytoplasm (white arrow heads) (after [16]); Bar 2 μm. (C) Time course of myelin phagocytosis and degradation (Po) and Galectin-3/MAC-2 protein (Gal-3) production. Phagocytosis and degradation of myelin result in the reduction of tissue content of the myelin specific molecule Po. Nerve segments located 5 millimeters distal to lesion sites were removed from wild-type mice at the indicated times and used to determine tissue levels of Po and Gal-3 by ELISA. Those are presented as percentage of their maximal values that are defined 100% (after [60]).

Nerve bundles in the PNS are mainly composed of axons, Schwann cells that enwrap those axons and further form myelin sheaths around many, fibroblasts that are scattered between nerve fibers, and vasculature that nourishes the PNS tissue (Figure 1A and 2A). Traumatic injury to PNS nerves produces abrupt tissue damage at the lesion site where physical impact occurred (Figure 1B). Then, nerve stumps that are located distal to lesion sites undergo the cellular changes that characterize Wallerian degeneration though they did not encounter the physical trauma directly. Amongst others, axons break-down, Schwann cells reject the myelin portion of their membranes, and bone-marrow derived macrophages are recruited and activated along with resident Schwann cells to remove degenerated axons and myelin (Figures 1C, D &1E and Figure 2B).

Lesions may be restricted in length; e.g. less than five millimeters in length, depending on how trauma is inflicted. On the other hand, distal nerve segments that undergo Wallerian degeneration and extend all the way towards their target tissues may range between several millimeters to many centimeters depending on species (e.g. mice versus humans) and site of trauma (e.g. near versus distant from innervated targets). When trauma produces complete transection of the PNS nerves, lesion sites include the gaps that are formed between proximal and distal nerve stumps.

Functional recovery depends on successful regeneration of the severed axons throughout distal nerve segments that undergo Wallerian degeneration. The most important determinant for good functional recovery in humans is prompt regeneration of the severed axons [7-10]. Notably, repair is often less successful in humans than it is in mice and rats. This discrepancy has been attributed to the delayed onset of axon destruction, the longer nerve segments that need to be cleared of degenerated myelin, and the longer distances that regenerating axons need to grow to reach their target tissues in humans. It is thought, therefore, that speeding Wallerian degeneration may improve functional recovery.

### Axon destruction and myelin disintegration

Species, axon diameter and length of the distal segment determine how fast axons break-down during normal Wallerian degeneration [11-13]. Fragmentation of axons is first detected by light microscopy 36 to 44 hours after nerve transection in mice and rats (Figure 1C), but only after about one week in baboons. Then, axon destruction may advance anterogradely at velocities ranging from about 10 to 24 mm/hour. However, freeze fracture studies reveal changes in the distribution of intramembranous particles in axons already 24 hours after the injury, and in Schwann cells that enwrap those axons even earlier - after 12 hours [14]. Disintegration of the myelin sheath, and Schwann cell proliferation and rearrangement into Bünger bands begin 2 days after injury [15]. The break-down of axons and myelin, along with other features of PNS Wallerian degeneration (see below), is delayed dramatically by 2 to 3 weeks in mutant Wld^s ^mice (formerly named Ola mice) [12,16,17]. Therefore, Wallerian degeneration in wild-type mice is defined here "normal" and in Wld^s ^mice "slow".

The molecular mechanisms that link between nerve injury at lesion sites and the destruction of axons during normal Wallerian degeneration have not been fully clarified; discussed in detail in [3,5,18]. The finding of the aberrant molecule that is composed of the N-terminal 70 amino acids of multiubiquitination factor Ube4b fused to NAD^+ ^synthesizing enzyme Nmnat1 in Wld^s ^mice led to the notion that isoform(s) of Nmnat, which are produced in neuronal cell bodies and transported anterogradely, protect axons by inhibiting a self-destructing mechanism [19-23]. In this context, depletion of Nmnat in axons consequent to cutting off supply from the cell body, as after nerve injury or knocking-out Nmnat, promotes axon destruction, and conversely, overexpression provides neuroprotection. It is further proposed that Nmnat dysfunction may underlie neuropathies that are not triggered by trauma, and that Nmnat-dependent signaling may be targeted to promote neuroprotection. It is unclear which product(s) of the Nmnat signaling cascade confer neuroprotection directly, and what is the nature of the self-destructing mechanism that Nmnat signaling inhibits.

The molecular mechanisms that link between nerve injury at lesion sites and myelin disintegration further distal during normal Wallerian degeneration have also not been entirely elucidated. However, the rapid and transient activation of the Erb2 receptor in Schwann cells by axon-derived neuregulin(s), which is detected 1 hour after the injury, may be involved [24]. It is unclear how injury initiates neuregulin-Erb signaling, how neuregulin-Erb signaling propagates anterogradely, and how, if at all, do Nmnat and neuregulin-Erb signaling cascades relate one to the other. Notably, neuregulin-Erb interactions may regulate both myelination and demyelination [25-31]. It has been suggested that BACE1 (β-amyloid precursor protein cleaving enzyme 1), which also cleaves neuregulin, regulates myelination and remyelination [32-34]. Further, BACE1 does not affect myelin disintegration but impedes clearance of degenerated myelin during Wallerian degeneration as BACE1 knock-out mice display faster clearance of myelin whereas time to onset of myelin and axon disintegration are not altered from normal [35].

### Degenerated myelin is harmful

Removal of degenerated myelin is critical for repair since PNS myelin contains molecules that inhibit regeneration of severed axons (e.g. MAG; myelin associated glycoprotein) [36-40]. Indeed, clearance of myelin, axon regeneration, and functional recovery are delayed considerably in Wld^s ^mice compared to those in wild-type mice [41-43]. Regeneration of severed axons in Wld^s ^mice is improved after knocking-out MAG even though myelin removal is still slow [40]. In accord, PNS myelin and MAG inhibit regeneration in-vitro [37-39]. The in-vitro inhibition of axon growth may not be detected depending on neuron identity (e.g. neonate versus adult) and whether adhesion or growth factors are present. These features may explain a report that PNS myelin is not inhibitory [44]. Further, contradictory results on CNS myelin associated inhibitors (e.g. Nogo, MAG and OMgp; oligodendrocyte myelin associated glycoprotein) have also been reported and further been explained through differences in experimental designs [45,46]. Nonetheless, most evidence indicates that myelin as whole structure (i.e. specialized membranous extensions of Schwann cells in PNS and oligodendrocytes in CNS) inhibits the regeneration of adult PNS and CNS axons; e.g. [47,48] and recent reviews [49-51].

The rapid clearance of degenerated myelin can also avert damage from intact axons and myelin after partial injury to PNS nerves where some but not all axons are axotomized by the impact (Figure 1; imagine that axon A is situated next to axon E). Here, degenerated myelin may activate the complement system to produce membrane attack complexes which, in turn, inflict damage to remaining nearby intact axons and myelin [52-54]. The rapid clearance of degenerated myelin may impede the production of membrane attack complexes and the damage they cause. Of note, complement activation has also beneficial effects since it advances macrophage recruitment and phagocytosis of degenerated myelin (see below).

### Schwann cells and macrophages are activated to scavenge degenerated myelin

Resident Schwann cells and recruited macrophages clear degenerated myelin in wild-type mice during normal Wallerian degeneration; [16,55,56] and Figure 2B. In-vivo experimental manipulations of macrophage depletion [57], which test clearance by Schwann cells without macrophages, and freeze-damaging nerves [16], which test clearance by recruited macrophages without Schwann cells, further indicate that each cell type can remove myelin in-vivo without the other. Schwann cells [16,58] and macrophages [59] can each scavenge myelin in-vitro as well.

The time course of myelin clearance was studied in detail in wild-type mice during normal Wallerian degeneration following a cut injury; [60] and Figure 2C. It begins 3 to 4 days after the injury and is completed after 12 to 14 days. Myelin destruction and removal are delayed considerably during slow Wallerian degeneration in Wld^s ^mice, as are axon destruction and macrophage recruitment [16,17,42,60].

The time course of myelin removal is determined by the kinetics of macrophage recruitment and the kinetics of the activation of macrophages and Schwann cells to scavenge degenerated myelin. Bone-marrow derived macrophages, which are scarce in intact PNS nerves of normal and Wld^s ^mice, accumulate at injury sites within hours after the trauma through ruptured vasculature and secondary to the rapid local production of cytokines and chemokines that attract macrophages to these sites; [61-63] and Figure 1B. The recruitment of macrophages during normal Wallerian degeneration is by diapedesis through vasculature that is structurally intact since it does not encounter physical trauma directly. It begins 2 to 3 days after a cut injury and it peaks at about 7 days [16,42,43,64,65]. In contrast, macrophage recruitment is delayed considerably in Wld^s ^mice during slow Wallerian degeneration. However, Wld^s ^macrophages invade freeze-damaged Wld^s ^PNS nerves promptly [16], suggesting that Wld^s ^macrophages can respond to chemotactic signals that freeze-damaged nerves produce, and further, that chemotactic signals are not upregulated during slow Wallerian degeneration, as indeed it was later shown [61] (see also below). The exact molecular mechanisms that link between the physical impact at lesion sites and macrophage recruitment to distal nerve segments during normal Wallerian degeneration are not fully understood. Yet, cytokines and chemokines that attract macrophages [61-63,66-68], MMPs (matrix metalloproteinases) [69-72], and complement [73-75] play roles (see below).

CR3 (complement receptor-3) and SRA (scavenger receptor-AI/II) have long been suggested to mediate phagocytosis of degenerated myelin by macrophages in context of trauma [59,73,76-80]. Recently, a role for FcγR (Fcγ receptor) and endogenous anti-myelin Abs has also been suggested [81]. Further, phagocytosis is augmented 2 folds and more after degenerated myelin activates the complement system to produce the complement protein C3bi which opsonizes myelin. Consequently, CR3 may bind to C3bi-opsonized myelin through C3bi and to unopsonized myelin directly. CR3 functions, therefore, both as a C3bi-opsonic and a non-opsonic receptor. SRA functions as a non-opsonic receptor that binds unopsonized myelin directly. However, SRA may also assist in the phagocytosis of C3bi-opsonized myelin since C3bi-opsonization does not block SRA binding sites on myelin. Altogether, CR3 contributes 2 to 3 folds more to myelin phagocytosis than SRA. Apart from complement, inflammatory cytokines TNFα (tumor necrosis factor-α) and IL (interleukin)-1β, which are produced during normal Wallerian degeneration, but not during slow Wallerian degeneration, also upregulate myelin phagocytosis by macrophages [63]. Of note, CR3 and SRA are similarly involved in myelin phagocytosis by CNS microglia.

Galectin-3/MAC-2 activates macrophages and Schwann cells to scavenge degenerated myelin (Appendix 1). Therefore, the time-course of Galectin-3/MAC-2 expression may reflect the kinetics of phagocytosis activation during Wallerian degeneration. Expression was studied in detail in the same wild-type and Wld^s ^mice in which myelin clearance and macrophage recruitment were examined; [16,60] and Figure 2C. Intact wild-type PNS nerves do not express detectable levels of Galectin-3/MAC-2. Expression is rapidly and transiently upregulated during normal Wallerian degeneration following cut injuries. Galectin-3/MAC-2 is first detected in Schwann cells 48 to 72 hours after injury, and then also in recruited macrophages. Notably, the onset of Galectin-3/MAC-2 expression precedes myelin clearance, expression is highest during the time period at which most of the degenerated myelin is removed, and expression is down-regulated after myelin clearance is completed. Galectin-3/MAC-2 is not expressed in intact Wld^s ^PNS nerves or during slow Wallerian degeneration, but is expressed in injured Wld^s ^PNS nerves at lesion sites where macrophages accumulate and phagocytose degenerated myelin. Thus, the occurrence and timing of Galectin-3/MAC-2 expression in cells that scavenge myelin are in accord with those of myelin clearance. The cytokine GM-CSF (granulocyte colony stimulating factor), which is produced during normal Wallerian degeneration, but dramatically less during slow Wallerian degeneration, upregulates the expression of Galectin-3/MAC-2 in macrophages, Schwann cells and the entire PNS nerve tissue [60,82].

Galectin-3/MAC-2 expression and the occurrence of myelin phagocytosis correlate in the CNS as they do in the PNS. CNS microglia that fail to phagocytose degenerated myelin in-vivo during CNS Wallerian degeneration do not express Galectin-3/MAC-2 [83]. In contrast, microglia that phagocytose degenerated myelin in-vivo during experimental allergic encephalomyelitis [84] and in-vitro [85] express Galectin-3/MAC-2.

### The cytokine network of Wallerian degeneration

**Figure 3:**
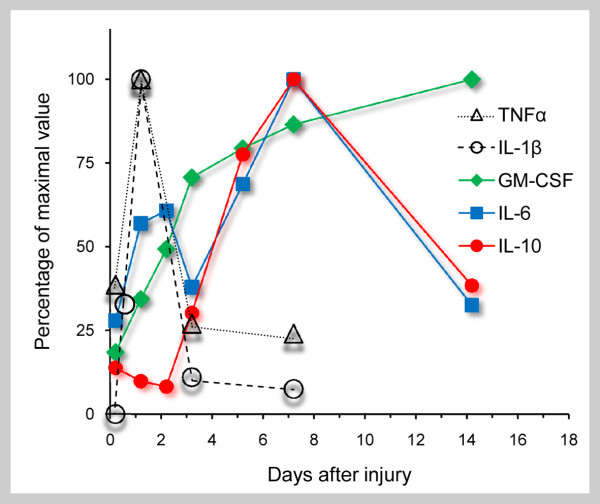
**The time course of cytokine protein secretion during normal Wallerian degeneration**. Nerve segments located 5 millimeters distal to lesion sites were removed from wild-type mice at the indicated times and used to condition medium with secreted cytokine proteins that were detected and quantified by ELISA. Values are presented as percentage of maximum secretion which is defined 100% (after [60,86]). The secretion of IL-1α is detected within 6 hours after the injury; not shown here since the method of detection was by a bioassay [87].

**Figure 4:**
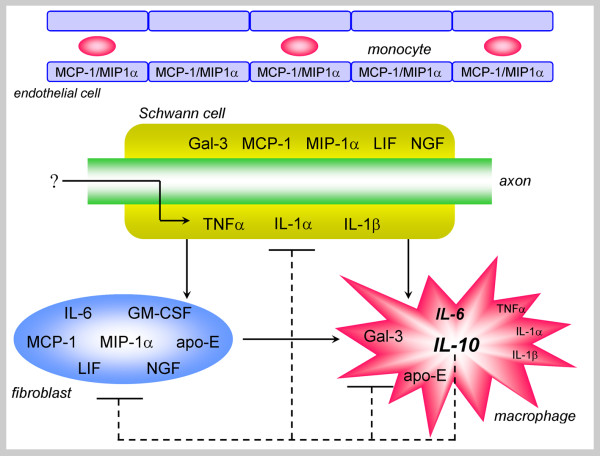
**The cytokine network of Wallerian degeneration**. Injury sets in motion the cytokine network of normal Wallerian degeneration. Intact myelinating Schwann cells enwrap intact axons and further express normally the inflammatory cytokines TNFα and IL-1α mRNAs and the TNFα protein. Traumatic injury at a distant site in the far left (not shown) induces the rapid upregulation of TNFα and IL-1α mRNAs expression and proteins production and secretion by Schwann cells within 5 hours. The nature of the signal(s) that are initiated at the injury site, travel down the axon, and then cross over to Schwann cells are not known (?). Concomitantly, Schwann cell derived TNFα and IL-1α induce resident fibroblasts to upregulate the expression of cytokines IL-6 and GM-CSF mRNAs and the production and secretion of their proteins within 2 to 5 hours after the injury. Inflammatory IL-1β mRNA expression and protein production and secretion are induced in Schwann cells with a delay of several hours. The expression of chemokines MCP-1/CCL2 and MIP-1α/CCL3 are upregulated by TNFα, IL-1β and IL-6 as of day 1 after the injury in Schwann cells, and possibly also in fibroblasts and endothelial cells. In turn, circulating monocytes begin their transmigration into the nerve tissue 2 to 3 days after the injury. Fibroblasts begin producing apolipoprotein-E (apo-E) and Schwann cells Galectin-3/MAC-2 (Gal-3) just before the onset of monocyte recruitment. Apolipoprotein-E and Galectin-3/MAC-2 may drive monocyte differentiation towards M2 phenotype macrophage which further produces apolipoprotein-E and Galectin-3/MAC-2. Macrophages efficiently produce IL-10 and IL-6 and much less TNFα, IL-1α, IL-1β. The anti-inflammatory cytokine IL-10, aided by IL-6, down-regulates productions of cytokines. Schwann cells and fibroblasts produce also LIF. Arrows indicate activation and broken lines down-regulation. Not all possible interactions and molecules produced are shown (e.g. autocrine interactions and the role of GM-CSF inhibitor); see text for additional information. The break-down of axons and myelin, and their phagocytosis are not illustrated here; see, however, Figure 1 and Figure 2.

PNS injury induces immune and non-immune cells to produce cytokines (Appendix 2) at and distal to lesion sites. Consequently, a cytokine network is set in motion in wild-type mice during normal Wallerian degeneration (Figure 3A and 4A). Cytokine mRNAs expression and detailed kinetic studies of cytokine protein production and secretion, along with the identification of the producing cells, were carried out after complete nerve transection in the same wild-type and Wld^s ^mice in which myelin clearance, macrophage recruitment and Galectin-3/MAC-2 expression were studied; see above and [60,63,82,86,87]. Findings suggest that timing and magnitude of cytokine production depend on the identity and spatial distribution in the PNS tissue of the non-neuronal cells that produce cytokines, and the timing of macrophage recruitment.

Resident Schwann cells normally express the mRNAs of the inflammatory cytokines TNFα and IL-1α, and the TNFα protein. Schwann cells that form close contacts with axons are the first amongst non-neuronal cells to respond to axotomy by rapidly upregulating the expression and production of TNFα and IL-1α mRNAs and proteins; the secretion of TNFα and IL-1α proteins is detected within 5 to 6 hours after injury. Schwann cells also express and produce IL-1β mRNA and protein, the secretion of which is detected between 5 to 10 hours after injury. This delayed expression and production of IL-β may be induced by the Schwann cell-derived TNFα, thus through an autocrine effect. Concomitantly, Schwann cell-derived TNFα and IL-1α induce nearby resident fibroblasts to express and further produce the mRNAs and proteins of cytokines IL-6 and GM-CSF, the secretion of which is detected within 2 to 5 hours after the injury. Of note, the highest levels of TNFα and IL-1β protein secretion are detected 1 day after the injury, thus before macrophage recruitment begins. IL-6 protein secretion is biphasic; the first phase peaks at day 2 just before macrophage recruitment begins, and the second peaks at day 7.

Inflammatory cytokines and chemokines (see below) advance the recruitment of blood-borne macrophages. Recruitment begins 2 to 3 days after the injury and peaks at about 7 days. The production and secretion of TNFα and IL1-β proteins is reduced while macrophages increase in number, suggesting that recruited macrophages produce little TNFα and IL1-β. Recruited macrophages produce and secrete IL-6 and IL-10 proteins, but little if any GM-CSF protein. The second phase of IL-6 production develops and then peaks at day 7 after the injury concomitant with the timing and magnitude of macrophage recruitment. The production and secretion of the anti-inflammatory cytokine IL-10 protein is induced in resident fibroblasts within 5 hours after injury, but levels are low and ineffective since nerve-resident fibroblasts are poor producers of IL-10, and Schwann cells do not produce IL-10. In contrast, recruited macrophages produce and secrete IL-10 protein effectively; levels increase and then peak at day 7 concomitant with the timing and magnitude of macrophage recruitment. Then, IL-10 gradually down-regulates the production of cytokines, bringing the cytokine network of normal Wallerian degeneration to conclusion 2 to 3 weeks after injury, which is after degenerated myelin has already been cleared. Of note, the production and secretion of GM-CSF protein is attenuated but not reduced during the second stage of normal Wallerian degeneration. However, at that time, a GM-CSF binding molecule that inhibits GM-CSF activity is produced [88].

Cytokines mRNAs expression was studied after crush injuries that are followed by axonal regeneration 4 to 7 days after the injury. In one study [72], the induction of TNFα and anti-inflammatory TGF-β1 mRNAs was biphasic; the first peaked at day 1 and the second at day 7 after crush. In other studies (summarized in [2]), a single phase of induction that peaked at day 1 after crush was detected for TNFα, IL-1β, IL-6 and IL-10 mRNAs. Evidently, discrepancies exist between the kinetics of cytokine proteins production and secretion following cut injuries and the kinetics of cytokine mRNAs expression following crush injuries. These may be due to the different paradigms of injuries used. Crush but not cut injuries enable regeneration and potential regulation of cytokine mRNA expression by the growing axons.

It is useful to characterize the profiles of production of cytokine proteins during the first and second phases of normal Wallerian degeneration; i.e. before and after macrophage recruitment. The first phase is characterized by the production of the inflammatory cytokines TNFα, IL-1α, IL1-β, GM-CSF and IL-6. The second phase is characterized by the production of IL-10, IL-6, and a GM-CSF inhibitor molecule, and furthermore, by the reduced production of TNFα and IL1-β. Therefore, the first phase is mostly inflammatory and the second is predominantly anti-inflammatory. Further, it is very likely that recruited macrophages are of the M2 phenotype which is involved in tissue repair (Appendix 3), since they produce high levels of IL-10 and IL-6, less TNFα and IL1-β, and little if any GM-CSF. Apolipoprotein-E [89,90] and Galectin-3/MAC-2 [91] can both direct the polarization of recruited macrophages towards the M2 phenotype. Apolipoprotein-E is produced and secreted by resident fibroblasts during normal Wallerian degeneration as of day 2 and later on also by macrophages [92,93] as is Galectin-3/MAC-2 (see above). Of note, both apolipoprotein-E and Galectin-3/MAC-2 are produced in Wld^s ^mice at injury sites but not during slow Wallerian degeneration.

A deficient cytokine network develops during slow Wallerian degeneration in Wld^s ^mice since the production of cytokine proteins is dramatically lower during slow Wallerian degeneration than it is during normal Wallerian degeneration even though the expression of cytokine mRNAs is upregulated [60,63,82,86]. In contrast, cytokine mRNAs are expressed and proteins produced in injured Wld^s ^PNS nerves at lesion sites concomitant with macrophage accumulation and activation to phagocytose myelin. The development of an efficient cytokine network during normal Wallerian degeneration versus a deficient cytokine network during slow Wallerian degeneration, along with other aspects of innate-immunity (e.g. macrophage recruitment and phagocytosis of degenerated myelin), highlight the inflammatory nature of normal Wallerian degeneration.

The observation that cytokine proteins are not produced during slow Wallerian degeneration even though the expression of their mRNAs is upregulated [63], suggests that cytokines mRNAs and proteins are differentially regulated during Wallerian degeneration, and furthermore, that mRNA expression does not necessarily indicate that the respective protein is produced. Therefore, it is useful to study both cytokine protein production and secretion along with cytokine mRNA expression.

### Chemokines, recruitment of macrophages and Wallerian degeneration

Chemokine MCP-1 (chemoattractant protein-1; known also as CCL2, C-C motif ligand 2) and MIP-1α (macrophage inflammatory protein-1α; known also as CCL3) promote the transmigration of monocytes across the endothelial cell wall of blood vessels (Figure 4). MCP-1/CCL2, which Schwann cells produce, is upregulated within hours after the impact at injury sites, and after 1 day at distal domains during normal Wallerian degeneration [6,61,62,66-68,94]. MCP-1/CCL2 production is induced by TNFα and IL-1β, which Schwann cells synthesize (see above), partly by signaling through TLRs (toll-like receptors). In Wld^s ^mice, MCP-1/CCL2 is produced at injury sites, but not further distal where slow Wallerian degeneration develops. Therefore, the occurrence and timing of MCP-1/CCL2 production are in accord with those of TNFα and IL-1β that induce them. These events further correlate with the occurrence and timing of macrophage recruitment that MCP-1/CCL2 promotes. Studies in non-neuronal tissues suggest the involvement of IL-6-dependent MCP-1/CCL2 production by fibroblasts [95], and TNFα and IL1-β-dependent production by endothelial cells [96]. Macrophage recruitment is also promoted by MIP-1α/CCL3 [68]. Studies in Schwann cell tumors and non-neural tissues suggest that Schwann cells, fibroblasts, endothelial cells and macrophages may produce MIP-1α/CCL3 upon activation by TNFα, IL-1α and IL-1β [96-98]. Recruitment is further aided by TNFα-dependent induction of MMP-9 (matrix metalloproteinase-9) that Schwann cells produce [69-72] and by complement [73-75].

### Immune inhibitory receptors and Wallerian degeneration

Innate-immune functions are regulated by the interplay and balance between activating and inhibitory signals; neither acts in an "all or none" fashion. Inhibition may be produced by a family of immune inhibitory receptors. SIRPα (signal-regulatory-protein-α; known also as CD172α and SHPS1) is a member of this family [99-102]. SIRPα is expressed on myeloid cells (e.g. macrophages and microglia) and some neurons, and is activated by its ligand CD47 (known also as IAP - integrin associated protein). CD47 is a cell membrane protein receptor that various cells express (e.g. red blood cells, platelets and some neurons). Cells that express CD47 down-regulate their own phagocytosis by macrophages after CD47 binds to SIRPα on phagocytes. CD47 functions, therefore, as a marker of "self" that protects cells from activated autologous macrophages by sending a "do not eat me" signal.

CD47 is expressed on myelin and the myelin-forming Schwann cells and oligodendrocytes, and furthermore, myelin down-regulates its own phagocytosis by macrophages and microglia through SIRPα-CD47 interactions [85]. CD47 may function, therefore, as a marker of "self" that protects intact myelin, Schwann cells and oligodendrocytes from activated macrophages in PNS and activated microglia and macrophages in CNS. This mechanism may be useful under normal conditions and while combating invading pathogens since it protects bystander intact myelin and myelin-forming cells from macrophages and microglia that are activated to scavenge and kill pathogens. However, the very same mechanism may turn harmful when faster removal of degenerating myelin is useful; e.g. as after traumatic axonal injury [7-10] (see above also). Therefore, normal Wallerian degeneration does not display the fastest possible rate of in-vivo myelin clearance.

### Neurotrophic factors and Wallerian degeneration

Peripheral nerve injury induces the production of neurotrophic factors by Schwann cells and fibroblasts during normal Wallerian degeneration. Neurotrophic factors are peptides that regulate, amongst others, neuronal survival, axon growth and synapse formation during normal development and during adulthood after traumatic PNS nerve injury and other neuropathologies. They exert their effects on axons after binding to their cognate receptors at nerve endings and/or after being transported retrogradely to neuronal cell bodies. This review is not aimed at discussing neurotrophic factors in detail. Nonetheless, nerve injury induced production of NGF (nerve growth factor), IL-6 and LIF (leukemia inhibitory factor) will briefly be reviewed to highlight how the innate-immune properties of normal Wallerian degeneration may regulate neurotrophic functions.

Among families of neurotrophic factors is the neurotrophin family. It consists of NGF, BDNF (brain derived neurotrophic factor), NT (neurotrophin)-3, and NT-4/5; their functions and mechanisms of action have been extensively reviewed elsewhere; e.g. [103-107]. The production of NGF, BDNF and NT-4 is upregulated during normal Wallerian degeneration [108-113]. Among these, NGF promotes neuronal survival and axon growth of sympathetic and subsets of sensory dorsal root neurons. Since these neurons send their axons through PNS nerves, they can interact with NGF that is produced during normal Wallerian degeneration as they regenerate. NGF mRNA expression is upregulated in two phases at the injury site and further distal to it; the first peaks within hours and the second 2 to 3 days after the injury. IL-1α, IL-1β and TNFα contribute to NGF mRNA upregulation in fibroblasts but not in Schwann cells. Of note, NGF mRNA and protein upregulations correlate only partly since only the second phase of mRNA expression is coupled with a corresponding upregulation in NGF protein production [108]. The upregulation NGF mRNA expression is prolonged after cut injuries but transient after crush injuries, suggesting that axons that regenerate after crush down-regulate NGF expression [110]. Further, the upregulation of NGF mRNA expression is impeded during slow Wallerian degeneration in Wld^s ^mice [42] as are IL-1β and TNFα protein productions [63].

IL-6 is a member of the IL-6 family that includes amongst others LIF and CNTF (ciliary neurotrophic factor) [104,114,115]. The production of IL-6 and LIF is upregulated during normal Wallerian degeneration; IL-6 by resident fibroblasts and recruited macrophages [63,86], and LIF by resident Schwann cells and fibroblasts [116,117]. Apart from being modulators of innate-immune functions, IL-6 [118-120] and LIF [121,122] also display neurotrophic properties by promoting neuronal survival and axon growth. Further, LIF may also function as a Schwann cell growth factor [123].

### Neuropathic pain and Wallerian degeneration

The innate-immune response of injury-induced Wallerian degeneration may also produce neuropathic pain; i.e. the development of spontaneous pain and/or painful sensation to innocuous stimuli. This review is not aimed at discussing neuropathic pain in detail, but to highlight its relationship to injury-induced Wallerian degeneration. In general, neuropathic pain develops in association with various pathologies through diverse mechanisms; recently reviewed in [124-126]. One class of mechanisms relates to the innate-immune properties of Wallerian degeneration as revealed by the observations that injury-induced neuropathic pain is delayed and reduced in Wld^s ^mice [127] and also in IL-6 deficient mice [128]. Further, neuropathic pain (also referred to as inflammatory pain) can be evoked by inflammation without injury [129-135]. IL-1β, TNFα, and NGF, which are produced during normal Wallerian degeneration, have been implicated. IL-1β and TNFα may sensitize intact axons to produce spontaneous activity and/or enhanced activity in response to mechanical and thermal stimuli. IL-1β and TNFα further induce the expression of NGF, which, in turn, sensitizes sensory nerve endings. This is mostly evident after partial PNS nerve injury where some but not all axons are traumatized (Figure 1; imagine that axon A is situated next to axon E). Therefore, delayed and reduced neuropathic pain in Wld^s ^mice may be explained, at least in part, by reduced productions of IL-6, IL-1β, TNFα, and NGF.

### Putting it altogether - orchestration is important

Successful functional recovery by regeneration is promoted by the removal of inhibitory degenerated myelin and production of neurotrophic factors. Innate-immune mechanisms that develop during normal Wallerian degeneration regulate both. Those, in turn, depend on the orchestrated interplay between Schwann cells, fibroblasts, macrophages, and endothelial cells and molecules they produce (Figure 4).

Intact Schwann cells are best suited amongst non-neuronal cells to "sense" and rapidly respond to the axotomy at remote sites by rapidly upregulating the expression and production of TNFα and IL-1α first, and IL-1β thereafter. This is made possible since Schwann cells form intimate contacts with axons, molecular machineries by which axons and Schwann cells communicate signals exists (e.g. neuregulin-Erb interactions), and intact Schwann cells further normally express TNFα and IL-1α, which enables their fast upregulation.

Schwann cell-derived TNFα, IL-1α and IL-1β induce adjacent resident fibroblasts to produce IL-6, GM-CSF and LIF within few hours after injury. Thereafter, TNFα, IL-1α, IL-1β and IL-6 induce the production of MCP-1/CCL2 and MIP1-α/CCL3 in Schwann cells, fibroblasts and endothelial cells. The two chemokines promote the transmigration of bone-marrow monocytes across structurally intact walls of blood vessels into the PNS nerve tissue. Consequently, the recruitment of monocytes begins 2 to 3 days after the injury, reaching highest numbers at about 7 days. Apolipoprotein-E and Galectin-3/MAC-2 that are produced before and during monocyte recruitment may help drive monocyte differentiation towards the M2 phenotype tissue macrophage.

Schwann cells and axons display minor structural changes 12 and 24 hours after the injury and profound disintegration 2 to 3 days after the injury. They then become amenable for scavenging by activated Galectin-3/MAC-2 expressing macrophages and Schwann cells; the onset of clearance is 3 to 4 days after the injury and completion is after 12 to 14 days. Indeed, there is a remarkable matching between setting-up the machinery for scavenging the degenerated myelin and its actual removal. Setting-up begins with the recruitment of macrophages and the activation of macrophages and Schwann cells to express Galectin-3/MAC-2 by fibroblast-derived GM-CSF before the onset of myelin clearance; most of the degenerated myelin is removed when activated Galectin-3/MAC-2^+ ^macrophages and Schwann cells reach highest numbers; activation (Galectin-3/MAC-2 expression) is down-regulated after degenerated myelin is removed.

Bringing the innate-immune response to conclusion is aided by the production of the anti-inflammatory cytokine IL-10 which TNFα, IL-1α and IL-1β induce in the recruited M2 phenotype macrophages. Effective IL-10 levels are reached 7 days after the injury when macrophage recruitment peaks. Then, IL-10 gradually down-regulates the production of cytokines; reaching lowest levels in about 2 to 3 weeks after injury, thus well after degenerated myelin is cleared. The GM-CSF inhibitor molecule and IL-6, due to its anti-inflammatory properties, help to down-regulate the production and activity of cytokines.

Wallerian degeneration further upregulates neurotrophic properties. The production of NGF is rapidly induced after the injury in Schwann cells and fibroblasts; in the latter by TNFα, IL-1α and IL-1β. Further, IL-6 and LIF function as neurotrophic factors as well as classical cytokines. Therefore, the development of some neurotrophic properties is tightly associated with the development of the innate-immune properties of normal Wallerian degeneration.

The failure to develop an efficient innate-immune response in slow Wallerian degeneration supports the view that innate-immunity plays critical roles in normal Wallerian degeneration and the restoration of function that follows. The innate-immune response of normal Wallerian degeneration depends on upregulating the production of TNFα and IL-1α proteins in Schwann cells. However, the production of these cytokine proteins is not upregulated during slow Wallerian degeneration even though the expression of their mRNAs is induced. In accord with the notion that TNFα and IL-1α proteins help putting the innate-immune properties of normal Wallerian degeneration in motion, the failure to upregulate their protein production during slow Wallerian degeneration impedes dramatically the development of an efficient innate-immune response, NGF production, and repair.

## Conclusion

Innate-immunity is central to injury-induced PNS Wallerian degeneration since innate-immune cells, functions and molecules are involved. Repair depends on an efficient innate-immune response that helps turning the PNS tissue into an environment that supports axon regeneration by removing inhibitory myelin and by upregulating neurotrophic properties. Recovery is poor when innate-immune mechanisms fail to develop. Therefore, the innate-immune mechanisms of Wallerian degeneration may be targeted to ensure successful functional recovery from trauma.

## Abbreviations

Apo-E: (apolipoprotein-E); BDNF: (brain derived neurotrophic factor); CCL2: (C-C motif ligand 2); CNS: (central nervous system); CR3: (complement receptor-3); FcγR: (Fcγ receptor); GM-CSF: (granulocyte colony stimulating factor); IL: (interleukin); LIF: (leukemia inhibitory factor); MAG: (myelin associated glycoprotein); MCP-1: (chemoattractant protein-1); MIP-1α: (macrophage inflammatory protein-1α); MMP: (matrix metalloproteinase); NGF: (nerve growth factor); NT: (neurotrophin); OMgp: (oligodendrocyte myelin associated glycoprotein); PNS: (peripheral nervous system); SIRPα: (signal-regulatory-protein-α); SRA: (scavenger receptor-AI/II); TNF: (tumor necrosis factor).

## Competing interests

The authors declare that they have no competing interests.

## Authors' contributions

SR wrote the manuscript

## Appendices

### Appendix 1: Galectin-3/MAC-2 activates myelin phagocytosis by macrophages and further promotes Schwann cells to scavenge myelin

Galectin-3, formally named MAC-2 [136], is a multifunctional β-galactoside binding protein and a member of the Galectin family of lectins; reviewed recently in [137-139]. It is present in the nucleus and cytoplasm of many cells, and it may also be secreted. Cytosolic Galectin-3/MAC-2 activates myelin phagocytosis in macrophages and microglia. Myelin phagocytosis by CR3 and SRA involves signaling through phosphatidylinositol 3-kinase (PI3K) [140,141]. PI3K is preferentially activated by K-Ras.GTP which Galectin-3/MAC-2 binds and stabilizes [137,142]. As a result, Galectin-3/MAC-2 enhances K-Ras.GTP-dependent functions. K-Ras.GTP/PI3K-dependent phagocytosis of degenerated myelin is similarly activated by Galectin-3/MAC-2 [143,144].

The molecular mechanisms that enable Schwann cells to scavenge their own degenerated myelin are unclear as Schwann cells do not express CR3, SRA or FcγR that mediate myelin phagocytosis in macrophages and microglia. However, Galectin-3/MAC-2 may be involved [16]. Intact myelinating Schwann cells do not express detectable levels of Galectin-3/MAC-2, but they do so as they internalize degenerated myelin in-vivo during normal Wallerian degeneration and in-vitro during in-vitro Wallerian degeneration; i.e. when intact nerves are moved to culture and so degenerate in the absence of recruited macrophages. Further, galactose and lactose, which inhibit binding to Galectin-3/MAC-2, impede the disintegration and internalization of myelin by Schwann cells. Therefore, secreted Galectin-3/MAC-2 is likely involved.

### Appendix 2: Cytokines are multi functional proteins

Cytokines are small proteins that regulate innate-immune functions in immune cells (e.g. phagocytosis and production of cytokines by macrophages). However, some cytokines further modulate immune and non-immune functions in non-immune cells (e.g. production of cytokines and nerve growth factor in fibroblasts), and others (e.g. IL-6 and LIF) also display neurotrophic properties (see text). Therefore, many cytokines are indeed multifunctional. While most cytokines function after being released from the producing cells, others may also function membrane-bound (e.g. TNFα). Further, cytokines may be divided into major classes. Inflammatory cytokines (e.g. TNFα, IL-1α, and IL-1β; also referred to as pro-inflammatory) promote the production of inflammatory mediators, and anti-inflammatory cytokines (e.g. IL-10) down-regulate the production of inflammatory mediators. Nonetheless, some cytokines (e.g. IL-6) display both inflammatory and anti-inflammatory properties [145]; see also review [146].

### Appendix 3: Tissue macrophages can differentiate into M1 and M2 phenotypes

M1 and M2 phenotypes are two extremes of a spectrum. The M1 phenotype is considered inflammatory since, amongst others, it produces the inflammatory cytokines TNFα, IL-1α and IL-1β, and is involved in killing pathogens. The M2 phenotype is considered anti-inflammatory since, amongst others, it produces the anti-inflammatory cytokine IL-10, it does not produce inflammatory cytokines or very little, and is involved in tissue remodeling and wound-healing. Both M1 and M2 phenotype macrophages produce IL-6, which is both inflammatory and anti-inflammatory in nature, and further function as phagocytes; reviewed in [147-152].
